# Targeted and untargeted metabolomics provide insight into the consequences of glycine‐N‐methyltransferase deficiency including the novel finding of defective immune function

**DOI:** 10.14814/phy2.14576

**Published:** 2020-09-19

**Authors:** Brandon J. Eudy, Caitlin E. McDermott, Xiuli Liu, Robin P. da Silva

**Affiliations:** ^1^ Department of Food Science and Human Nutrition University of Florida Gainesville FL USA; ^2^ Department of Pathology, Immunology and Laboratory Medicine University of Florida Gainesville FL USA

**Keywords:** aryl hydrocarbon receptor, fibrosis, NAD, one‐carbon metabolism, tryptophan

## Abstract

Fatty liver disease is increasing along with the prevalence of obesity and type‐2 diabetes. Hepatic fibrosis is a major health complication for which there are no efficacious treatment options available. A better understanding of the fundamental mechanisms that contribute to the accumulation of fibrosis is needed. Glycine‐N‐methyltransferase (GNMT) is a critical enzyme in one‐carbon metabolism that serves to regulate methylation and remethylation reactions. GNMT knockout (GNMT^‐/‐^) mice display spontaneous hepatic fibrosis and later develop hepatocellular carcinoma. Previous literature supports the idea that hypermethylation as a consequence of GNMT deletion contributes to the hepatic phenotype observed. However, limited metabolomic information is available and the underlying mechanisms that contribute to hepatic fibrogenesis in GNMT^‐/‐^ mice are still incomplete. Therefore, our goals were to use dietary intervention to determine whether increased lipid load exacerbates steatosis and hepatic fibrosis in this model and to employ both targeted and untargeted metabolomics to further understand the metabolic consequences of GNMT deletion. We find that GNMT mice fed high‐fat diet do not accumulate more lipid or fibrosis in the liver and are in fact resistant to weight gain. Metabolomics analysis confirmed that pan‐hypermethylation occurs in GNMT mice resulting in a depletion of nicotinamide intermediate metabolites. Further, there is a disruption in tryptophan catabolism that prevents adequate immune cell activation in the liver. The chronic cellular damage cannot be appropriately cleared due to a lack of immune checkpoint activation. This mouse model is an excellent example of how a disruption in small molecule metabolism can significantly impact immune function.

## 1 INTRODUCTION

The rise in prevalence of obesity mirrors the rise in incidence of nonalcoholic fatty liver disease (NAFLD), nonalcoholic steatohepatitis (NASH), hepatic fibrosis and cirrhosis (Younossi et al., 2016). Accumulation of fibrosis is enhanced in individuals with NAFLD and histological fibrosis stage is the best predictor of long‐term outcomes in patients who develop NASH (Angulo et al., 2015). Unfortunately, there are currently no efficacious therapies available to treat hepatic fibrosis. Fibrosis naturally occurs after cellular damage, but normal immune function allows for fibrosis regression. The accumulation of extracellular matrix over time is, therefore, due to an imbalance in fibrogenesis and fibrosis regression (Schuppan, Surabattula, & Wang, 2018). Theoretically, the accumulation of extracellular matrix could be the result of chronic lesions that overwhelm the capacity for repair or as a result of a defect in the tissue repair. The latter has not been extensively investigated in models of hepatic fibrosis. Recent work has demonstrated that circulating monocytes are recruited to the liver where they can differentiate into macrophages and help to regulate inflammation and tissue repair in the context of fibrosis (Ju and Tacke, (2016)). Therefore, we sought to investigate the role of the immune system in a model of hepatic fibrosis.

GNMT knockout (GNMT^‐/‐^) mice were generated by Luka et al. and it was demonstrated that they spontaneously develop fibrosis and multifocal hepatocellular carcinoma (Luka, Capdevila, Mato, & Wagner, 2006; Martinez‐Chantar et al., 2008). Following this finding, it was hypothesized that elevated AdoMet was necessary for development of steatosis. This was supported by the observation that feeding a methionine deficient diet normalized AdoMet levels and the liver phenotype (Martinez‐Una et al., 2013; Zubiete‐Franco et al., 2016). From this it was suggested that aberrant methylation was part of the mechanism of fibrosis development in these mice. Indeed, it was demonstrated that enhanced flux through phosphatidylethanolamine N‐methyltransferase (PEMT) produced phosphatidylcholine that is converted to triglyceride in GNMT^‐/‐^ mouse liver (Martinez‐Una et al., 2013). Although it is not clear that PEMT dependent triglyceride (TG) synthesis contributes significantly to hepatic steatosis nor whether lipotoxicity is a significant contributing factor to liver damage in GNMT^‐/‐^ mouse liver (Silva, Kelly, Al Rajabi, & Jacobs, 2014). We hypothesized that lipotoxicity would not be a major factor in GNMT^‐/‐^ mouse liver fibrosis since there is abundant PC synthesis that would prevent toxic lipid accumulation. We decided to assess this using a high‐fat diet challenge. Natural Killer (NK) cell activation was required for development of significant hepatic fibrosis in GNMT^‐/‐^ mice (Gomez‐Santos et al., 2012) and more recently cell death‐mediated by TNF‐related apoptosis‐inducing ligand (TRAIL) secreting NK cells were necessary for fibrosis in this mouse model (Fernandez‐Alvarez et al., 2015). However, the underlying mechanisms that cause enhanced cell death were not identified. Moreover, the idea that GNMT^‐/‐^ mice may have inadequate tissue repair, and hence, poor fibrosis regression have not been explored as potential contributors to progression of liver damage in GNMT^‐/‐^ mice.

We now demonstrate that hepatic steatosis and fibrosis in GNMT^‐/‐^ mice are not exacerbated after long‐term consumption of high‐fat diet (HFD) and that GNMT^‐/‐^ mice are resistant to HFD‐induced weight gain suggesting that lipotoxicity is not a major contributor to liver damage in this model. Furthermore, we utilized both targeted and untargeted metabolomics to comprehensively assess the metabolic profile in GNMT^‐/‐^ mice. From this we have identified significant impacts on serotonin, aryl hydrocarbon and nicotinamide (NAM) metabolism and signaling in GNMT^‐/‐^ mice. Finally, we show that these metabolic consequences of GNMT deficiency impair the function of platelets, reduce the number of circulating immune cells and inhibit inflammatory pathways that are critical for tissue repair.

## 2 METHODS

### 2.1 Animals

Male C57BL/6J and GNMT^‐/‐^ mice were weened at 21 days and housed in ventilated cages with corn cob bedding and maintained on a 10:14‐hr light/dark cycle. All mice were maintained on ad libitum standard chow diet until the 8 weeks of age. At 8 weeks wild‐type (WT) and GNMT^‐/‐^ mice were provided ad libitum* *access to either a control of HFD for 8 weeks. Diets were made using Envigo basal diet (without 20% oil by weight) mix #TD88232 with the following additions: control diet consisted of 1% lard, 1% canola oil, 3% corn oil, and 15% corn starch by weight; HFD contained 15% lard, 2% canola oil, and 3% corn oil by weight. Body weights were measured at the beginning of the study, and every third day after for all 8 weeks of the feeding study. Food intake and food efficiency were determined using chow diet in order to maintain a more accurate account of food intake that was not possible using the powdered experimental diet. All study protocols were approved by the Institutional Animal Care and Use Committee of the University of Florida and were in accordance with the Guide for the Care and Use of Laboratory Animals.

### 2.2 Liver TG

Frozen liver tissues were cut and homogenized for 2 × 10 s in 9 volumes of phosphate‐buffered saline (PBS). Lipids were extracted using a modified Folch lipid extraction (Folch, Lees, & Sloane Stanley, 1957). Briefly, 3 volumes of chloroform:methanol (2:1) was added to 1 volume of sample homogenate in PBS and vortexed for 30 s. Samples were centrifuged for 5 min at 2000 RPM. The lower phase of the samples was transferred to a new tube that was heated to 50°C and placed under a stream of nitrogen gas for ~40 min until completely dried. Samples were then placed on ice. Lipids were dissolved in 2‐propanol and quantified using the TG‐SL kit from Sekisui^TM^ and glycerol as a standard. Absorbance values were read at 505 nm and 660 nm using a Spectramax (Molecular Devices, Sunnyvale, CA). The 660 nm reading was subtracted from the 505 nm reading and the lipid concentration was calculated using the standard curve.

### 2.3 Serotonin, AdoMet, and AdoHcy

Plasma and platelet serotonin was measured using a modification of the method of Yoshitake, Kehr, Todoroki, Nohta, & Yamaguchi (2006). Briefly, plasma or platelet‐rich plasma samples were deproteinized using 2 volumes of acetonitrile and vortexed vigorously for 20 s. Samples were centrifuged, the supernatant was transferred to new tubes and dried under vacuum. To the dried extract 200 μl of derivatization reagent comprised of 60% water, 40% methanol with 0.5 of M benzimidazole, 10 mM of potassium hexacyanoferrate(III) and cyclohexylaminopropanesulfonic acid (CAPS). The reaction was heated to 50°C for 20 min. Products were separated using a C18 BEH Acquity Column and a Waters H‐class UHPLC and detected using a fluorescence detector at 345 nm excitation and 480 nm emission. AdoMet and AdoHcy were determined using UPLC as previously described (Jacobs et al., 2005).

### 2.4 Metabolomics

Liver samples that were snap‐frozen in liquid nitrogen were sent to the Southeastern Center for Integrated Metabolomics (SECIM) headquartered at the University of Florida. Untargeted metabolomics were performed using LC‐MSMS using a high‐resolution Orbitrap Mass spectrometer (Thermo, Gainesville, FL). Targeted metabolomics of purine and pyrimidine nucleotides was performed using single reaction monitoring after UPLC separation.

### 2.5 Cytokine analysis

Cytokine analysis was performed in both liver tissue using ProcartaPlex^TM^ Multiplex Immunoassay and a Luminex 200^TM^. The measured cytokines were GM‐CSF, G‐CSF, M‐CSF, IFN‐У, IL‐2, IL‐6, IL‐10, CXCL5, MCP‐1, MIP‐1α, sRANKL, VEG‐F, and RANTES. The plasma cytokines measured were G‐CSF, GM‐CSF, M‐CSF, IL‐1β, IFN‐У, IL‐4, IL‐10, IL‐17a, IP‐10, Leptin, sRANKL, RANTES, VEGF‐A, and CXCL5. Assays were performed as per the manufacturer's instruction (Thermo Fisher, Carlsbad, CA).

### 2.6 Flow cytometry

For white blood cell analysis, mixed blood was collected from heart puncture into EDTA‐treated vacuum tubes (BD biosciences, Franklin Lakes, NJ). Erythrocytes were lysed in hypotonic ammonium‐chloride buffer for 10 min with gentle agitation at 4°C, and then, centrifuged at 250× *g* for 10 min at 4°C. The supernatant was removed, and pellet was washed twice with cold PBS. The pellet was then resuspended in PBS containing 5 mM of EDTA and 0.5% (w/v) bovine serum albumin (FACS buffer). Purified white blood cells were probed 30 min with the following antibodies: Anti‐CD45‐APC (Cell Signaling Technologies, Danvers, MA), anti‐Ly6G‐FITC (Sigma‐Aldrich, St. Louis, MO), and anti‐CD64‐PE (Thermo‐Fisher). Samples were washed twice with FACS buffer, resuspended in 300 μl of FACS buffer and analyzed immediately.

For platelet analysis, 200 µl of whole blood was collected from the inferior vena cava and added to tubes containing 40 ul of acid‐citrate‐dextrose buffer at 30°C and a portion of blood was aliquoted for counting. Blood was diluted 100x in cold FACS buffer and mixed gently. Diluted whole blood was probed with anti‐CD9‐APC (Thermo‐Fisher) for 30 min. Samples were washed twice with FACS buffer, resuspended in 300 μl of FACS buffer and analyzed immediately.

The Accuri C6 flow cytometer and software (BD Biosciences) was used for all data acquisition and analysis.

### 2.7 Histology

At time of sacrifice, liver sections were cut and placed in 10% of phosphate‐buffered formalin for histology. Samples were processed at the Molecular Pathology Core at the University of Florida for processing. Paraffin‐embedded liver sections were sliced, deparaffinized, and stained with picrosirius red using a standard protocol. Images were captured using a Nikon Eclipse Ti2 fluorescent microscope and data were quantified using Nikon Elements version 4.6.

### 2.8 Western blotting

Mouse livers were homogenized in ice‐cold RIPA buffer containing protease and phosphatase inhibitors (Thermo‐Fisher), and then, diluted in laemmli buffer containing 5% of β‐mercaptoethanol before being heated at 95°C for 5 min. Proteins were resolved by SDS‐PAGE before being transferred to a nitrocellulose membrane. Membranes were probed with primary antibodies targeting the aryl hydrocarbon receptor (AhR) (Cell Signaling Technologies), cleaved caspase‐3 (Cell Signaling Technologies), Ly6G/6C (Thermo‐Fisher), glycoprotein VI (GPVI) (Millipore, Burlington, MA), 5‐hydroxytrytamine receptor 2B (5‐HT_2B)_ (Novus Biologicals, Littleton, CO), indoleamine 2,3‐dioxygenase (IDO2) (Thermo‐Fisher), nuclear factor kappa‐light‐chain‐enhancer of activated B cells (NFkB) p65 subunit (Cell Signaling Technologies), phospho‐NFkB p65 subunit (Cell Signaling Technologies), Poly [ADP‐ribose] polymerase 1 (PARP‐1) (Proteintech, Rosemont, IL), protein disulfide isomerase (PDI, Cell Signaling Technologies), or vinculin (Cell Signaling Technologies) overnight before being probed with an appropriate secondary antibody for 1 hr. The Clarity electrochemiluminescent substrate kit and ChemiDoc MP imaging system (BioRad, Hercules, CA) were used for detection and image acquisition.

### 2.9 qPCR

Real‐time quantitative PCR was used to measure mRNA abundance of selected gene targets. Total RNA was extracted from THP‐1 macrophages as per the manufacturer's protocol using the Qiagen RNeasy Plus Mini Kit (Qiagen, Germany). Isolated RNA was checked for purity and concentration using a nanophotometer (Implen, Germany). cDNA was synthesized using the Applied Biosystems High‐Capacity Reverse Transcription Kit (Applied Biosystems, Foster City, CA). Primers corresponding to each target gene were designed using the Universal Probe Library (Roche, Switzerland). RT‐qPCR was performed using the PowerUp SYBR Green master mix (Applied Biosystems). Amplification data were normalized to the housekeeping gene, cyclophilin B, and results were quantified using the ΔΔCT method.

### 2.10 Statistical analysis

Statistics was performed using GraphPad Prism 8 for all measurements, with the exception of metabolomics. Untargeted data were analyzed using the MetaboAnalyst open source online software. Otherwise data are expressed at mean ± *SEM*. One‐way analysis of variance was used to determine significance in all multigroup analyses. *P* value of <.05 indicates significance.

## 3 RESULTS

### 3.1 GNMT^‐/‐^ mice were resistant weight gain

**Figure 1:**
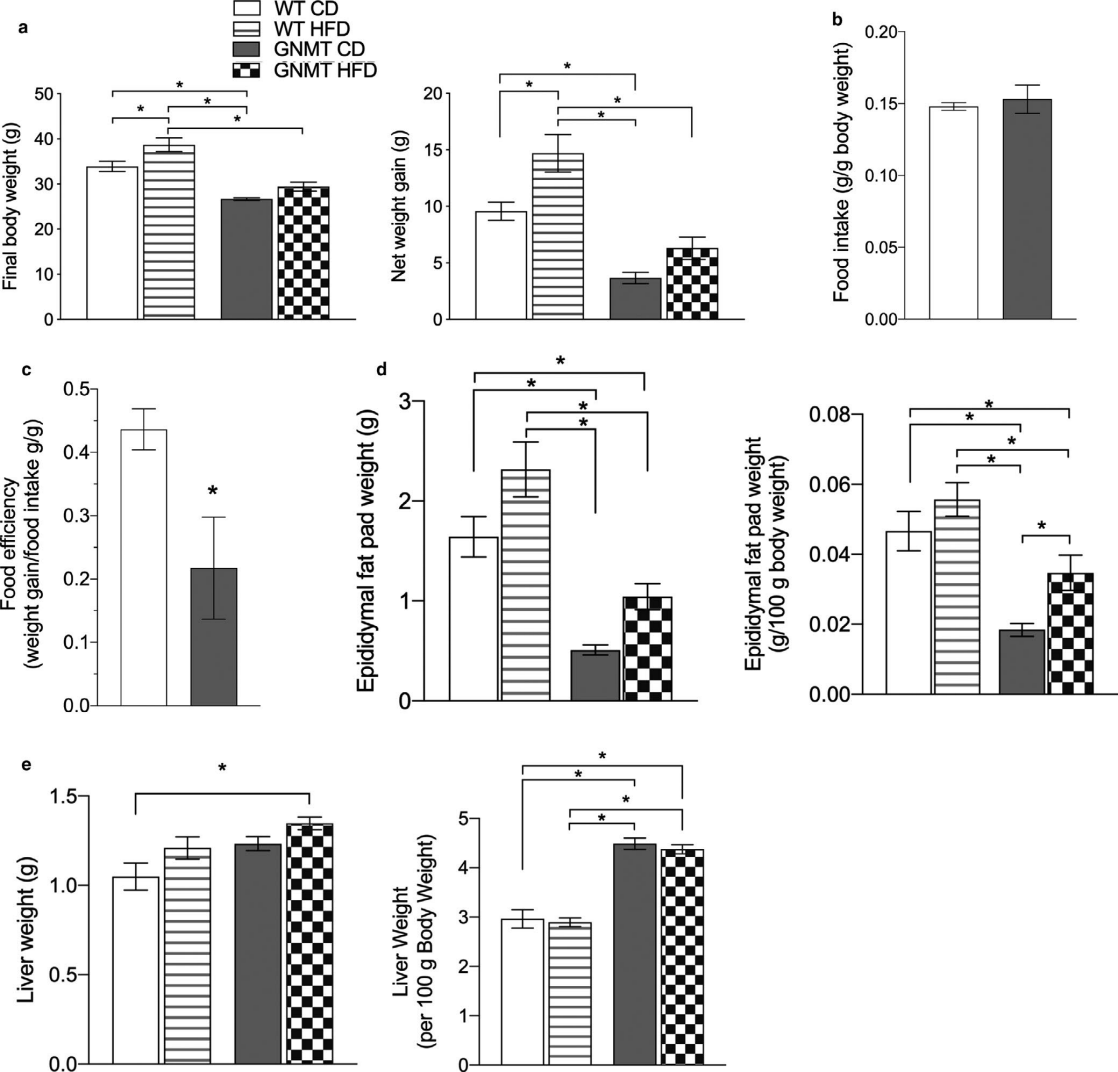
GNMT^‐/‐^ mice are resistant to body weight gain when fed HFD. Final body weight and net weight gain over 8 weeks in grams (a). Food intake normalized to body weight (chow diet) (b). Food efficiency reported as grams body weight gained per gram daily food intake (chow diet) (c). Epididymal fat pad weight in grams and normalized to body weight (d). Liver weight in grams and liver weight normalized to body weight (e). White bars represent wild‐type (WT) mice fed control diet (CD); White bars with stripes represent WT mice fed HFD; Grey bars represent glycine‐N‐methyltransferase knockout (GNMT^‐/‐^) mice fed CD; Checkered bars represent GNMT^‐/‐^ mice fed HFD. Data are presented as mean ± *SEM*. An asterisk represents a significant difference (*p* < .05, *n* = 6–10)

GNMT^‐/‐^ mice were previously reported to have increased liver triglyceride content and it was proposed that enhanced hepatic PEMT activity induces TG synthesis and lipid droplet formation (Martinez‐Una et al., 2013). However, only chow diets were utilized in these experiments and the impact of a HFD was not explored. After 8 weeks of feeding a 20% of fat diet, we serendipitously discovered that GNMT^‐/‐^ mice were resistant to HFD‐induced weight gain (Figure 1a). GNMT^‐/‐^ mice ate the same proportional amount of food as WT mice and consequently had a significantly lower food efficiency (Figure 1b,c). Furthermore, GNMT^‐/‐^ mice did not increase adipose tissue mass like WT mice adding only 40% of the epididymal fat pad mass per gram body weight on the normal fat diet and only 50% of WT on the HFD at the end of 8 weeks (Figure 1d). Liver weights were not significantly different in GNMT^‐/‐^ mice compared to WT mice except those on the HFD but were significantly larger when corrected for body weight (Figure 1e). Hepatic TG were not significantly different between any of the mice (Figure 1f). The lack of a difference in liver TG was likely due to the use of a purified diet in this study as compared to chow diets used in previous studies.

### 3.2 HFD diet consumption did not affect the severity of fibrosis

**Figure 2:**
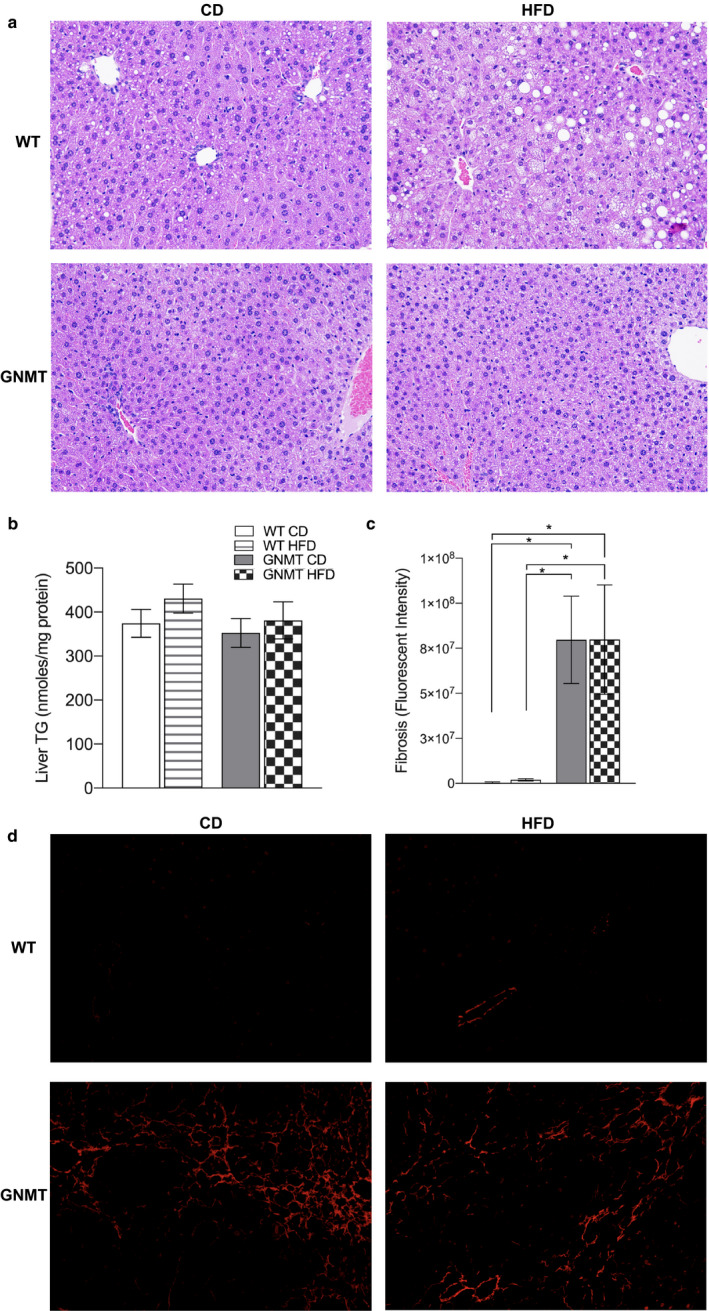
HFD does not exacerbate steatosis or fibrosis in livers of GNMT^‐/‐^ mice. Hematoxalin and Eosin staining of liver sections (a). Liver triglyceride (TG), nanomoles per mg protein (b). Quantification of picrosirius red fluorescence from liver, fluorescence intensity (c). Fluorescent images of picrosirius red staining of liver (d). White bars represent wild‐type (WT) mice fed control diet (CD); White bars with stripes represent WT mice fed HFD; Grey bars represent glycine‐N‐methyltransferase knockout (GNMT^‐/‐^) mice fed CD; Checkered bars represent GNMT^‐/‐^ mice fed HFD. Data are presented as mean ± *SEM*. An asterisk represents a significant difference (*p* < .05, *n* = 8–10)

Morphologically GNMT^‐/‐^ mouse livers had a higher proportion of microvesicular steatosis and fewer large lipid droplet when compared to WT controls in both diet treatments (Figure 2a). Hepatic TG was not different in the liver of any of the mice fed either control or HFD (Figure 2b). Picrosirius red staining showed significantly more fibrosis in GNMT^‐/‐^ mice when compared to controls and this was confirmed by image analysis of red staining (Figure 2c,d). Histological scoring was performed by a liver pathologist and showed significantly more fibrosis in GNMT^‐/‐^ mice but no difference in overall NASH score (Table 1). In addition, 8 weeks of HFD treatment did not influence histological scoring significantly.

**Table 1:** Histology scoring for liver damage

Measurement	WT CD	WT HFD	GNMT CD	GNMT HFD
Steatosis (0–3)	1.7 ± 0.8	2.0 ± 0.8	1.7 ± 1.2	1.6 ± 1.0
Lobular Inflammation (0–4)	1.0 ± 1.3	1.1 ± 0.6	1.4 ± 1.4	1.4 ± 1.1
Ballooning Score (0–1)	0.1 ± 0.4	0 ± 0	0.2 ± 0.4	0.2 ± 0.4
Apoptosis (0–2)	0.4 ± 0.8	0.8 ± 0.9	0.9 ± 1.0	1.0 ± 0.8
Fibrosis (0–4)	0.9 ± 1.5^a^	0.1 ± 0.4^a^	1.5 ± 1.8^b^	1.9 ± 1.5^b^
NASH Score	4.1 ± 4.2	4.0 ± 1.7	5.7 ± 5.5	6.1 ± 4.4

a a = 0.0363.

b b = 0.0034.

### 3.3 Untargeted and targeted metabolomics reveal significant alterations in tryptophan metabolism GNMT^‐/‐^ mice

Pathway analysis of untargeted metabolomics revealed an interesting and logical metabolic profile in GNMT^‐/‐^ mice (Table 2). The pathway with the most hits was purine metabolism and a number of other pathways that interact with purine metabolism. Several pathways of amino acid metabolism appeared in the top 10 pathways including histidine and tryptophan. Nicotinamide metabolism was in the top 10 pathways with the most significant hits. In addition, folate and energy metabolism were also among the most significantly affected pathways.

**Table 2:** Pathway analysis of untargeted metabolomics from liver of GNMT^‐/‐^ mice

	Pathway total	Hits.total	Hits.sig	Expected	FET
Purine metabolism	66	39	12	8.0187	0.073218
Arginine and proline metabolism	37	21	6	4.4953	0.23502
Arginine biosynthesis	14	10	5	1.7009	0.033047
Histidine metabolism	16	12	5	1.9439	0.073415
Alanine, aspartate, and glutamate metabolism	28	18	5	3.4019	0.28907
Tryptophan metabolism	41	28	5	4.9813	0.69782
Nicotinate and nicotinamide metabolism	15	12	4	1.8224	0.2089
Folate biosynthesis	24	12	4	2.9159	0.2089
Pyrimidine metabolism	39	19	4	4.7383	0.55459
Aminoacyl‐tRNA biosynthesis	22	20	4	2.6729	0.59879
Butanoate metabolism	15	9	3	1.8224	0.26593
Glyoxylate and dicarboxylate metabolism	31	14	3	3.7664	0.56024
Cysteine and methionine metabolism	33	16	3	4.0093	0.65726
Glycine, serine, and threonine metabolism	31	20	3	3.7664	0.80306
Synthesis and degradation of ketone bodies	5	3	2	0.60748	0.10537
Porphyrin and chlorophyll metabolism	27	4	2	3.2804	0.18319
D‐Glutamine and D‐glutamate metabolism	6	6	2	0.72897	0.3491
Propanoate metabolism	19	7	2	2.3084	0.42864
beta‐Alanine metabolism	21	8	2	2.5514	0.50277

**Figure 3:**
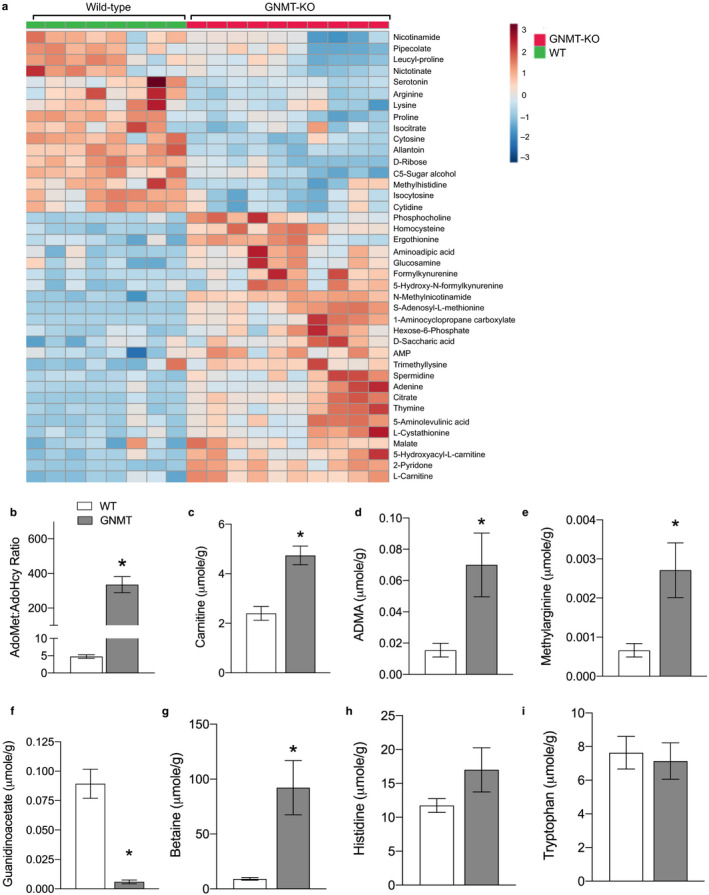
Untargeted and targeted metabolomics reveals altered amino acid metabolism and hypermethylation in GNMT^‐/‐^ mice. Heat map of top 40 differential metabolites from in liver of glycine‐N‐methyltransferase knockout (GNMT^‐/‐^) mice compared to wild‐type (WT) mice determined by hierarchical cluster analysis (a). The ratio of S‐adenosylmethionine (AdoMet) to S‐adenosylhomocysteine (AdoHcy) (b). Bar graphs from targeted metabolomics of one‐carbon metabolism in liver: carnitine (c), asymmetric dimethylarginine (ADMA) (d), methylarginine (e), guanidinoacetate (f), betaine (g), histidine (h), and tryptophan (i). White bars represent WT mice; Grey bars represent GNMT^‐/‐^ mice. Data are presented as mean ± *SEM*. An asterisk represents a significant difference (*p* < .05, *n* = 6–10)

To tease out the largest changes in metabolites a heat map with the top 40 metabolites was generated using hierarchical cluster analysis (Figure 3a). The largest differences between WT and GNMT^‐/‐^ mice were observed in metabolites of AdoMet, NAM, tryptophan, carnitine, lysine, nucleotides, carbohydrate, and the Kreb's (TCA) cycle. Many of these metabolic changes indicated enhanced methylation and were confirmed using a targeted metabolomics platform of one‐carbon metabolism (Figure 3b‐i). AdoMet and a number of its metabolites were significantly increased in GNMT^‐/‐^ mice. Carnitine and its metabolites trimethyllysine and 5‐hydroxyacyl carnitine were elevated in GNMT^‐/‐^ mice and the lysine catabolic products 5‐aminolevulinic acid, aminoadipic acid (increased), and pipecolate (reduced) indicating enhanced methylation and increased overall catabolism of lysine. Tryptophan metabolites from both the kynurenine and 5‐hydroxytryptamine (serotonin) pathways were significantly altered in GNMT^‐/‐^ mice. Serotonin was significantly reduced in the liver and there were significant increases in formylkynurenine and 5‐hydroxy‐formylkynurenine. Significant increases in 1‐methylnicotinamide and 2‐pyridone indicated that there was enhanced methylation of NAM in GNMT^‐/‐^ mice. Further, reduced nicotinate and ribose indicated that there may be significant alterations in nucleotide and NAD metabolism in GNMT^‐/‐^ mice.

**Figure 4:**
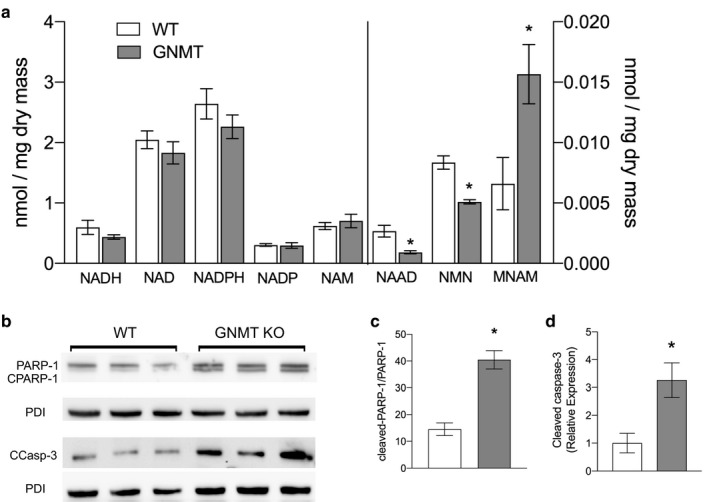
Targeted metabolomics show GNMT^‐/‐^ mice have altered nicotinamide metabolism. Nicotinamide metabolites in liver presented as nmole per mg dry liver tissue. MNAM = 1‐methylnicotinamide, NAAD = nicotinic acid adenine dinucleotide, NAM = nicotinamide, NMN = nicotinamide mononucleotide (a). Western blots of Poly [ADP‐ribose] polymerase 1 (PARP‐1) and cleaved caspase‐3 (CCASP‐3) in liver. Protein disulfide isomerase (PDI) was used as a loading control (b). PARP‐1 activation denoted as ratio of cleaved to full‐length PARP‐1 (c). Cleaved caspase‐3 expression normalized to PDI (D). White bars represent wild‐type (WT) mice; Grey bars represent glycine‐N‐methyltransferase knockout (GNMT^‐/‐^) mice. Data are presented as mean ± *SEM*. An asterisk represents a significant difference (*p* < .05, *n* = 4–10)

Previously, it was shown that feeding GNMT^‐/‐^ mice the methyl acceptor NAM as a sink for methyl groups normalized AdoMet levels and prevented fibrosis (Varela‐Rey et al., 2010). However, as well as serving as a methyl acceptor, NAM can also contribute to the NAD pool through the salvage pathway and thereby spare the need for de novo synthesis from tryptophan. We now demonstrate that there is indeed enhanced methylation of a large number of methyl acceptors in the liver including NAM under basal conditions with no added methyl acceptors (Figure 4a). Hepatic nucleotides indicate increased methylation of NAM and depletion of nicotinamide adenine dinucleotide precursors. In addition, increased PARP1 cleavage indicates that there is a need to conserve NAD in the liver and significant stress on this central metabolic pathway (Figure 4b,c). NAD can be synthesized from the kynurenine pathway via quinolinate, but accumulation of formylkynurenine coupled with the reduced folate‐mediated remethylation likely means that there is reduced capacity for conversion of formylkynurenine to kynurenine that precludes de novo NAD synthesis. In addition, there is increased hepatic caspase‐3 cleavage indicating that apoptosis is enhanced in GNMT mice, confirming previous reports (Fernandez‐Alvarez et al., 2015). Taken together, these data indicate that GNMT^‐/‐^ mice have impaired ability to furnish necessary de novo NAD that leads to cellular stress and apoptosis in the liver.

### 3.4 GNMT^‐/‐^ mice have impaired serotonin metabolism and platelet function

**Figure 5:**
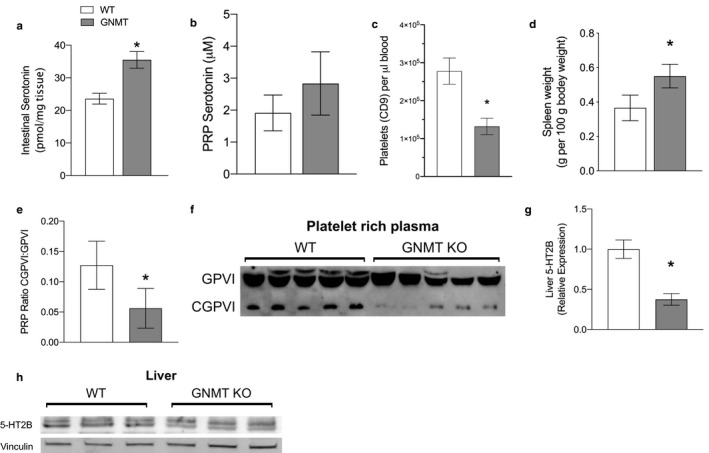
GNMT^‐/‐^ mice show perturbed systemic serotonin handling. Intestinal serotonin concentration as pmole per mg tissue (a). Platelet‐rich plasma (PRP) serotonin concentration as µM (b). Platelet count reported as CD9 + cells per µl whole blood (c). Spleen weight relative to body weight (d). Ratio of cleaved glycoprotein VI (GPVI) to full‐length GPV in platelet‐rich plasma (e). Western blot showing full‐length and cleaved GPVI in platelet‐rich plasma (f). 5‐hydroxytryptamine receptor 2B (5‐HT_2B_) protein expression in liver normalized to vinculin (g). Western blot showing 5‐HT_2B_ expression in the liver with Vinculin as loading control (h). White bars represent wild‐type (WT) mice; Grey bars represent glycine‐N‐methyltransferase knockout (GNMT^‐/‐^) mice. Data are presented as mean ± *SEM*. An asterisk represents a significant difference (*p* < .05, *n* = 4–10)

Depletion of serotonin in the liver suggested that intestinal serotonin metabolism is altered in these mice. Indeed, intestinal serotonin concentrations were elevated in the GNMT KO mice suggesting either impaired release, enhanced synthesis, or higher density of enterochromaffin cells in the intestine (Figure 5a). Serotonin content in platelet‐rich plasma (PRP) was not different between WT and GNMT^‐/‐^ mice (Figure 5b). However, GNMT^‐/‐^ mice had significantly reduced platelet counts in blood (48% of WT) (Figure 5c) indicating that net platelet serotonin was reduced. Platelets are sequestered in the spleen of subjects with liver disease and we observed significant splenomegaly in GNMT^‐/‐^ mice (Figure 5d). To assess the activation of platelets in GNMT^‐/‐^ mice we measured the cell surface collagen receptor GPVI. GPVI is normally cleaved by metalloproteinases during activation upon binding collagen but we observed significantly less proportional GPVI cleavage (40% of WT) in western blots from PRP (Figure 5e,f). Serotonin is released upon platelet activation, and therefore, we measured the serotonin (5‐HT_2B_) receptor expression (lower band) in liver which was lower in GNMT^‐/‐^ mice (30% of WT) (Figure 5g,h).

### 3.5 GNMT^‐/‐^ mouse livers recruit immune cells but are missing key immune checkpoints

**Figure 6:**
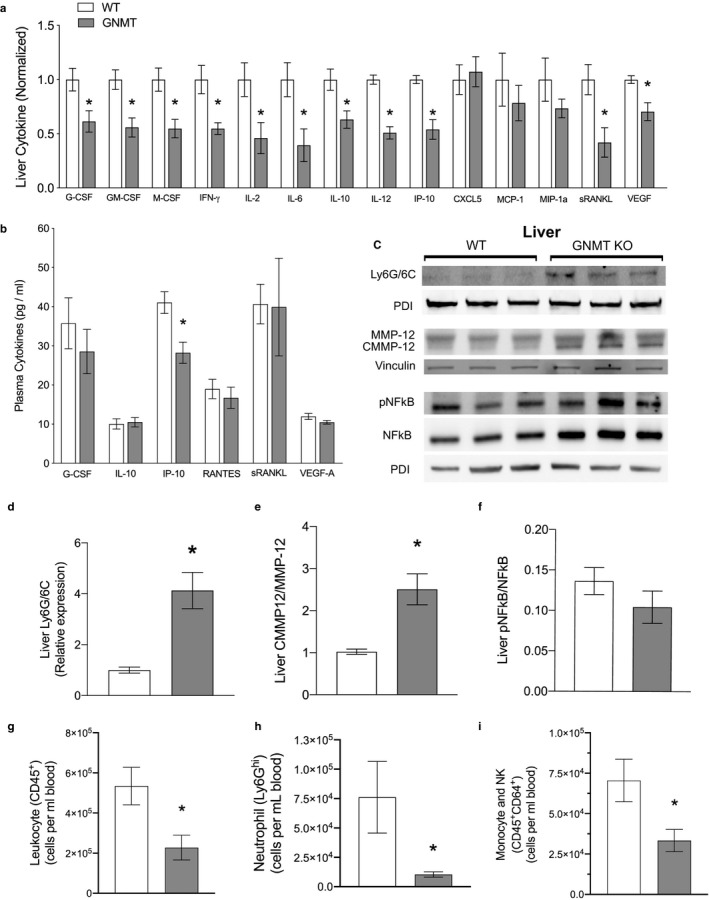
GNMT^‐/‐^ show reduced cytokine content in hepatic tissue despite significant immune infiltration to liver. Concentrations of C‐X‐C Motif Chemokine Ligand 5 (CXCL5, LIX), granulocyte colony stimulating factor (G‐CSF), granulocyte‐macrophage colony stimulating factor (GM‐CSF), interferon‐gamma (IFN‐γ), interleukin‐10 (IL‐10), interleukin‐12 (IL‐12), interleukin‐2 (IL‐2), interleukin‐6 (IL‐6), interferon (IFN)‐γ inducible protein (IP‐10), monocyte chemoattractant protein‐1 (MCP‐1), macrophage colony stimulating factor (M‐CSF), macrophage inflammatory protein‐1 alpha (MIP‐1α), receptor activator of nuclear factor kappa‐Β ligand (sRANKL), and vascular endothelial growth factor (VEGF‐A) in liver normalized to wild‐type (WT) mice (a). Concentrations of G‐CSF, IL‐10, IP‐10, regulated on activation, normal T cell expressed and secreted (RANTES), sRANKL, and VEGF‐A in plasma reported as picogram per ml (b). Representative western blot of Ly6G/6C, nuclear factor kappa‐light‐chain‐enhancer of activated B cells (NFκB) and macrophage elastase (MMP‐12) in liver with Protein disulfide isomerase (PDI) and Vinculin as a loading control (c). Hepatic Ly6G/6C protein expression normalized to PDI (d). MMP‐12 activation in liver presented as cleaved MMP‐12 relative to full‐length MMP‐12 (e). NFκB activation denoted as ratio of phosphorylated NFκB p65 subunit relative to total NFκB p65 (f). Leukocyte count in whole blood denoted as CD45^+^ cells per ml of blood (g). Neutrophil count in whole blood denoted as Ly6G^hi^ expressing cells in CD45^+^ cell population per ml of blood (h). Monocyte and NK cell count in whole blood denoted as CD45^+^CD64^+^ cells per ml blood (i). White bars represent WT mice; Grey bars represent glycine‐N‐methyltransferase knockout (GNMT^‐/‐^) mice. Data are presented as mean ± *SEM*. An asterisk represents a significant difference (*p* < .05, *n* = 4–10)

GNMT^‐/‐^ mice have reduced hepatic cytokine content consistent with impaired immune cell activation (Figure 6a). Of the 14 cytokines only four were above detection in plasma of WT and GNMT^‐/‐^ mice with only a significant reduction in circulating IP‐10 in GNMT^‐/‐^ mice which was 70% of that in WT mice (Figure 6b). Western blots showed increased expression of Ly6G/6C in the livers of GNMT^‐/‐^ mice (Figure 6c,d). Macrophage elastase (MMP‐12) and its cleaved active form (MMP‐12) were both elevated in GNMT^‐/‐^ mice (Figure 6c,e). No relative increase in phospho‐NFκB to total NFκB p65 expression (Figure 6c,f) indicating that the immune cells were no more active in the GNMT^‐/‐^ mouse liver. Leukocytopenia was observed in GNMT^‐/‐^ mice; flow cytometry for markers of total leukocytes, monocytes/NK cells and neutrophils in blood were significantly decreased by 43, 47, and 14%, respectively, in GNMT^‐/‐^ mice compared to WT controls (Figure 6g‐i). Together, these results demonstrate there is a clear recruitment of immune cells in the livers of GNMT^‐/‐^ mice but that there is not a classic inflammatory response in these mice.

**Figure 7:**
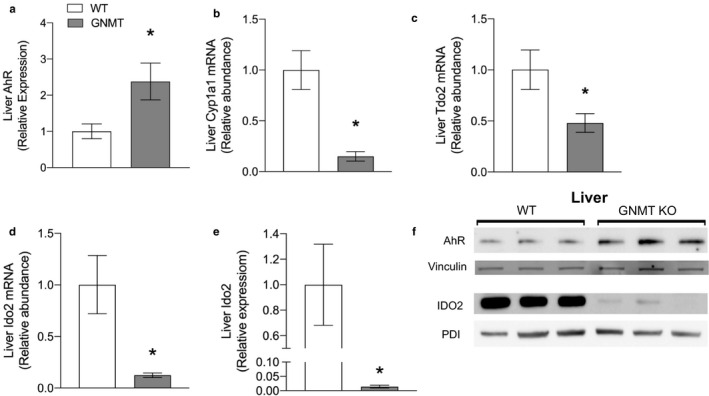
GNMT^‐/‐^ mice have reduced aryl hydrocarbon receptor activation and decreased expression of rate limiting enzymes of kynurenine pathway in liver. AhR protein expression in liver normalized to vinculin (a). Cytochrome P450, family 1, subfamily A, polypeptide 1 (Cyp1a1) mRNA abundance in liver (b). Tryptophan 2,3‐dioxygenase‐2 (Tdo2) mRNA abundance in liver (c). Ido2 abundance in liver (d). Indoleamine 2,3‐Dioxygenase 2 (Ido2) protein expression in liver normalized to PDI (e). Western blot showing aryl hydrocarbon receptor (AhR) and Ido2 expression in liver with Vinculin and Protein disulfide isomerase (PDI) as loading controls (f). White bars represent wild‐type (WT) mice; Grey bars represent glycine‐N‐methyltransferase knockout (GNMT^‐/‐^) mice. Data are presented as mean ± *SEM*. An asterisk represents a significant difference (*p* < .05, *n* = 4–6)

Given the striking alterations in tryptophan metabolism that coincide with a disruption in immune function, we investigated key signaling molecules related to metabolism of this amino acid to explain these observations. Hepatic AhR expression was increased in GNMT^‐/‐^ mice (240% of WT) suggesting that there was a deficit in signaling through this receptor in the liver (Figure 7a). This was confirmed when we found that the transcript for the AhR target Cytochrome P450, family 1, subfamily A, polypeptide 1 (*Cyp1a1)* was significantly lower in GNMT^‐/‐^ mice (18% of WT) (Figure 7b). The mRNA for tryptophan 2,3‐dioxygenase (Tdo2) was also significantly reduced in GNMT^‐/‐^ mice (48% of WT) and IDO2 expression was almost completely abolished in GNMT^‐/‐^ mice at the level of both protein and mRNA.

## 4 DISCUSSION

The diet modifications that we employed made no difference to the hepatic phenotype of GNMT^‐/‐^ mice. Therefore, we conclude that GNMT^‐/‐^ mice have at the very least a similar capacity to move TG through the liver and are not susceptible to hepatic fat accumulation due to an increased dietary TG load. Instead, we found that the GNMT^‐/‐^ mice were resistant to HFD‐induced obesity that we observed in the GNMT^‐/‐^ mice. This gross phenotype is likely multifactorial. GNMT is indeed expressed in the brain and GNMT^‐/‐^ mice have been shown to display hyperactivity and schizophrenic like behavior (Yang et al., 2012). In addition, there is some evidence that GNMT^‐/‐^ mice may be insulin resistant; this was more pronounced in female mice (Liao et al., 2016) but we do not have evidence of this in the current study. Although plasma insulin concentration is not always indicative of insulin signaling it is of note that circulating insulin was not different between GNMT^‐/‐^ and WT mice (data not shown). It is tempting to speculate that central nervous system serotonin or monoamine metabolism may be altered in GNMT^‐/‐^ mice. Monoamine uptake inhibitors that have been used to treat depression have shown significant weight loss in both mice and humans (Bello & Liang, 2011). In addition, there has been recent evidence that GNMT^‐/‐^ mice have enhanced flux through some methylation reactions in the liver (Hughey et al., 2018) and this is supported by our current findings. This hypermethylation phenotype in GNMT^‐/‐^ mice could enhance catechol‐O‐methyltransferase activity in the brain and thereby interfere with catecholamine signaling. In addition, GNMT deficiency could influence N‐methyl‐D‐aspartate signaling in the brain through modulation of glycine content (Yang et al., 2012).

Previously it had been proposed that GNMT^‐/‐^ mice had enhanced PEMT activity that promoted lipid droplet formation, stimulating hepatic lipid accumulation, and hence, liver damage (Martinez‐Una et al., 2013). We do agree that PEMT activity is increased in GNMT^‐/‐^ mice but do not find that GNMT^‐/‐^ mice are more susceptible to hepatic lipid accumulation when consuming HFD. GNMT^‐/‐^ mice did appear to have microvesicular steatosis in the liver when compared to WT mice but this was not quantified. Finally, the HFD did not significantly influence the level of hepatic fibrosis in GNMT^‐/‐^ mice. Thus, it would appear that the liver damage is likely not mediated by impaired lipid metabolism in this model. Instead, we provide evidence that GNMT^‐/‐^ mice have impaired nicotinamide metabolism with increased PARP‐1 activation suggesting that altered nucleotide metabolism is contributing to liver damage. GNMT^‐/‐^ mice have been reported to have increased DNA damage due to augmented folate metabolism (Wang et al., 2014) that thereby increases the need for PARP activity and demand for NAD^+^ as a substrate. We propose that NAD^+^ catabolism is greatly exacerbated by increased and aberrant methylation of nicotinamide and that this net increase in demand for NAD^+^ biosynthesis cannot be met due to reduced catabolism of tryptophan through the kynurenine‐quinolinate pathway. Cells that become damaged and deplete in NAD^+^ undergo apoptosis that contributes to the observed hepatic phenotype.

We now present the novel finding that GNMT^‐/‐^ mice have impaired platelet function. Platelet derived serotonin is essential for liver regeneration (Lesurtel et al., 2006) and most likely explains the impaired liver regeneration observed in GNMT^‐/‐^ mice after partial hepatectomy (Varela‐Rey et al., 2009). Serotonin receptor 5‐HT_2B_ expression is reduced in GNMT^‐/‐^ mice and this receptor has previously been shown to stimulate proliferation of hepatocytes and hepatic stellate cells (Ruddell et al., 2006). Release of serotonin is an initial response of activated platelets and our finding of increased platelet serotonin is indicative on reduced platelet activation. Moreover, there was reduced cleavage of GPVI in PRP. GPVI is a collagen binding receptor expressed on the surface of platelets that is cleaved by two members of the A desintegrin and metalloproteinase (ADAM) family as a crucial component of platelet activation (Bender et al., 2010). The reason behind reduced GPVI cleavage and platelet activation in GNMT^‐/‐^ mice is not yet clear but it is most likely due to impaired immune function that results in deficiency in appropriate activation of metalloproteinases.

GNMT^‐/‐^ mice clearly have more infiltration of macrophages as indicated by Ly6G/6C and MMP‐12 expression. Increased MMP‐12 secretion is indicative of increased phagocytosis of hepatocyte debris by macrophages (Ramachandran et al., 2012). However, MMP‐12 has been shown to inhibit infiltration of neutrophils into tissues and dampen inflammation through cleavage of a number of key immunoregulatory proteins (Bellac et al., 2014). MMP‐12 has previously been shown to cleave the Fc region of IgG’s that prevents binding to the Fc receptors on macrophages; thereby preventing lymphocyte‐mediated macrophage activation (Banda, Clark, & Werb, 1983). MMP‐12 expression has been linked to hepatic fibrosis through the attenuation of IL‐13‐dependent induction of MMP‐2, −9, and −13 in liver (Madala et al., 2010). In addition, TNF‐related apoptosis‐inducing ligand (TRAIL) has been established as a necessary component of fibrogenesis in GNMT^‐/‐^ mice (Fernandez‐Alvarez et al., 2015) and more recently it has been shown that the C‐terminal portion of MMP‐12 induces TRAIL‐mediated cell death in tumors cells (Dandachi et al., 2017). Thus, we conclude that there is infiltration of macrophages to clear apoptotic cell debris and that MMP‐12 expression may be important mediators of hepatic fibrosis in GNMT^‐/‐^ mice.

Despite having increased macrophage infiltration, GNMT^‐/‐^ mice actually have decreased expression of inflammatory cytokine and are deficient in several circulating innate immune cell types. This is particularly surprising given the clear fibrotic liver damage. However, we demonstrated a lack of NFκB phosphorylation in GNMT^‐/‐^ mouse liver that corresponded with decreased AhR‐mediated transcription and low expression of hepatic cytokines. AhR plays a major role in stimulating cytokine transcription and AhR deficient mice display an abnormal liver phenotype from birth, with microvesicular steatosis, alterations in extramedullary hematopoiesis (Schmidt, Su, Reddy, Simon, & Bradfield, 1996) and fibrosis (Andreola et al., 2004). Therefore, we conclude that reduced AhR signaling is at least partially responsible for the lack of immune signaling, and hence, accumulation of hepatic fibrosis in GNMT^‐/‐^ mice. Tryptophan metabolites are ligands for AhR and we found significantly lower expression of the rate limiting enzymes, TDO and IDO2 in the kynurenine pathway in GNMT^‐/‐^ mice. We postulate that decreased production or a redistribution of kynurenine metabolites decreases AhR signaling.

The preceding metabolite of kynurenine is formylkynurenine which requires tetrahydrofolate (THF) as an acceptor for removal of the formyl group. Metabolomics analysis revealed the increased concentrations of formylkynurenine was one of the most significant alterations and pathway analysis revealed a number of metabolites in tryptophan metabolism had been altered. We suspect is due to deficit in flux through methionine synthase that is a consequence of the massive increase in AdoMet concentration in GNMT^‐/‐^ mouse livers (Luka et al., 2007; Wang, Chen, Lin, Liu, & Chiang, 2011). The lack of outlets for one‐carbon units may contribute to the downregulation of tryptophan catabolism and an accumulation of formylkynurenine. A low methionine diet has been shown to prevent the hepatic phenotype in GNMT^‐/‐^ mice by lowering AdoMet (Martinez‐Una et al., 2013) and thereby reintroducing homocysteine remethylation, freeing up THF for metabolism of formylkynurenine. Moreover, hypermethylation of nicotinamide and increased PARP1 activity place more demand on the kynurenine‐quinolinate pathway of NAD^+^ synthesis, further depleting kynurenine metabolites. Similar to our observations in GNMT^‐/‐^ mice, IDO2 knockout mice do not initiate inflammation in tissues and display reduced levels of cytokines including TNFα, IL‐6, and IFN‐γ in ear tissue after inflammatory stimulus (Metz et al., 2014). These findings demonstrate the crucial nature of tryptophan metabolism in the regulation of immune processes that have implications in liver repair, fibrosis, and by extension hepatocellular carcinoma.

In summary, we find that increased dietary fat does not impact liver damage in GNMT^‐/‐^ mice and that these mice are in fact resistant to weight gain. Metabolomic analysis revealed significant perturbations in tryptophan and nicotinamide metabolism in GNMT^‐/‐^ mice. In addition, GNMT^‐/‐^ mice have dysfunctional platelets and altered hepatic serotonin signaling that prevent normal activation of immune response and tissue regeneration. While this study has identified dysfunctional platelet phenotype and hepatic serotonin signaling further research is necessary to determine the mechanisms that are responsible for these observations. Finally, deficient AhR signaling and increased demand for de novo NAD + biosynthesis are consistent with impair immune function and increased apoptosis that are observed in liver of GNMT^‐/‐^ mice. We propose that the sum of these impairments in metabolism, and consequently immune function, contribute significantly to enhanced tissue damage and the inability to repair the damage in GNMT^‐/‐^ mouse livers. We recognize that other pathways are most certainly involved in these observation and that further research in these areas is warranted.

## CONFLICT OF INTEREST

The authors have no conflict of interest to declare.

## AUTHOR CONTRIBUTIONS

B.J.E and C.E.M were primarily responsible for animal husbandry, tissue processing, fluorescence microscopy, cytokine analysis, liver TG analysis, data analysis, wrote portions of the methods section, and provided manuscript revisions. B.J.E was responsible for all flow cytometry, western blot, qPCR, assisted in preparation of tissues for HPLC analysis and did additional data analysis. X.L. was responsible for pathological analysis of liver tissues and provided some revisions to the final manuscript. R.D.S was responsible for study design, surgical procedures, HPLC analysis, all of the metabolomic data analysis, data analysis, figure composition, and writing of the manuscript.

## ETHICAL STATEMENT

This article is original work that does not involve human subjects. All animal experimental protocols were approved by the Institutional Animal Care and Use Committee of the University of Florida and were in accordance with the Guide for the Care and Use of Laboratory Animals.
